# Reliably estimating prevalences of atopic children: an epidemiological study in an extensive and representative primary care database

**DOI:** 10.1038/s41533-017-0025-y

**Published:** 2017-04-13

**Authors:** David H. J. Pols, Mark. M. J. Nielen, Joke C. Korevaar, Patrick J. E. Bindels, Arthur M. Bohnen

**Affiliations:** 1 Department of General Practice, Erasmus MC, University Medical Center Rotterdam, Rotterdam, The Netherlands; 2 NIVEL, Netherlands Institute for Health Services Research, Utrecht, The Netherlands

## Abstract

Electronic health records stored in primary care databases might be a valuable source to study the epidemiology of atopic disorders and their impact on health-care systems and costs. However, the prevalence of atopic disorders in such databases varies considerably and needs to be addressed. For this study, all children aged 0–18 years listed in a representative primary care database in the period 2002–2014, with sufficient data quality, were selected. The effects of four different strategies on the prevalences of atopic disorders were examined: (1) the first strategy examined the diagnosis as recorded in the electronic health records, whereas the (2) second used additional requirements (i.e., the patient had at least two relevant consultations and at least two relevant prescriptions). Strategies (3) and (4) assumed the atopic disorders to be chronic based on strategy 1 and 2, respectively. When interested in cases with a higher probability of a clinically relevant disorder, strategy 2 yields a realistic estimation of the prevalence of atopic disorders derived from primary care data. Using this strategy, of the 478,076 included children, 28,946 (6.1%) had eczema, 29,182 (6.1%) had asthma, and 28,064 (5.9%) had allergic rhinitis; only 1251 (0.3%) children had all three atopic disorders. Prevalence rates are highly dependent on the clinical atopic definitions used. The strategy using cases with a higher probability of clinically relevant cases, yields realistic prevalences to establish the impact of atopic disorders on health-care systems. However, studies are needed to solve the problem of identifying atopic disorders that are missed or misclassified.

## Atopic disorders: Clinical information improves prevalence estimates

The prevalence of atopic disorders in children can be more reliably calculated by incorporating clinical information with diagnosis data. Researchers in the Netherlands, led by David Pols from the Erasmus University Medical Center Rotterdam, examined the electronic health records of more than 660,000 children, aged 0 to 18, from a Dutch primary care database to determine the number of cases of atopic eczema, asthma, and allergic rhinitis. They looked for diagnosed children who also had at least two relevant clinical consultations and at least two relevant prescriptions. This strategy helps correct for the problem of overestimation, because it doesnot assume that a child, once diagnosed, will have an atopic disorder for life. However, other methods are still needed to identify cases that are missed or misclassified in the health database.

## Introduction

The rising prevalence of atopic disorders in children are an important global health problem.^1, 2^ Atopy is a (genetic) predisposition toward developing allergic hypersensitivity. The clinical manifestation of atopy is allergy. However, not all allergies are atopic. In this study, the word ‘‘atopic’’ refers to this genetically mediated predisposition, resulting in the clinical diagnosis by a general practitioners (GPs) of eczema, asthma, and allergic rhinitis. In many countries, primary care professionals, e.g., family doctors/GPs, diagnose and treat these atopic children. In the Netherlands, GPs, are in the frontline of the health-care system, are freely accessible, and use uniform coding systems for recording diagnosis and prescriptions. In principle, all non-institutionalized residents in the Netherlands are registered in a general practice, even if they do not visit the GP. Therefore, the electronic health records (EHR) stored in primary care databases contain valid information about the epidemiological denominator, making it a potentially important source of epidemiological data.

A meta-analysis based on questionnaires in the ‘‘open population’’, including children of all ages (0–18 years), showed average one-year worldwide prevalences for eczema, asthma, and rhinoconjunctivitis of 7.9%, 12.0%, and 12.7%, respectively.^3^ However, the accuracy of data obtained from a questionnaire depends on various items, including the accuracy and knowledge of the responders, and the definitions used by the researcher.^4^ When comparing ‘‘open population’’ data with data obtained from the EHRs of general practices, lower annual prevalences for eczema, asthma, and rhinoconjunctivitis were found, ranging (on average) from 1.8–9.5%, 3.0–6.5%, and 0.4–4.1%, respectively.^5^ Since these diagnoses are based on the assessment of a physician, these data could potentially form a more specific epidemiological source. Unfortunately, the annual prevalences of atopic disorders in general practice databases vary considerably;^5^ moreover, since these differences cannot be fully explained by country or year of study, this variation needs further consideration. Part of this variation might be explained by the fact that GPs often work with a ‘‘probability diagnosis’’ that inevitably creates a risk of misclassification, which could result in either over- or underestimation. Other possible explanations could be a variation in clinical knowledge and skills of the GP. Furthermore, there might also be some coding difficulties, when coding diseases in EHRs.

Some studies using primary care data have presented life-time cumulative prevalences;^6–9^ the prevalences found for eczema, asthma, and rhinoconjunctivitis ranged (on average) from 7.2–36.5%, 4.2–22.9%, and from 1.0–11.4%, respectively. However, the question arises as to what extent these *life-time* cumulative prevalences provide relevant information compared with *annual* point prevalences, knowing that these disorders are not always chronic.

To establish the impact of atopic disorders on health-care systems and their related costs, a more accurate estimation is required of the prevalence of atopic disorders derived from general practice databases. This study investigates the risk of misclassification, which could either result in overestimation or underestimation of atopic disorders. The results for annual point prevalence vs. life-time cumulative prevalence were compared using four different strategies using an extensive and representative primary care database.

## Results

### Patient selection

A total of 660,512 eligible children (aged 0–18years) were derived from the The Netherlands Institute for Health Services Research-Primary Care Database (NIVEL-PCD (period 2002–2014)). Of these, 24,477 (3.7%) children did not pass the data quality checks (Appendix 1) and 157,959 (23.9%) children were excluded because they had less than 3 years of follow-up. The final study group included 478,076 children, of whom 51.1% were male. Mean age of the children when entering the NIVEL-PCD was 7.2 (SD 6.0) years: mean follow-up time was 6.6 (SD 4.7) years.

### Prevalence of atopic eczema

**Fig. 1:**
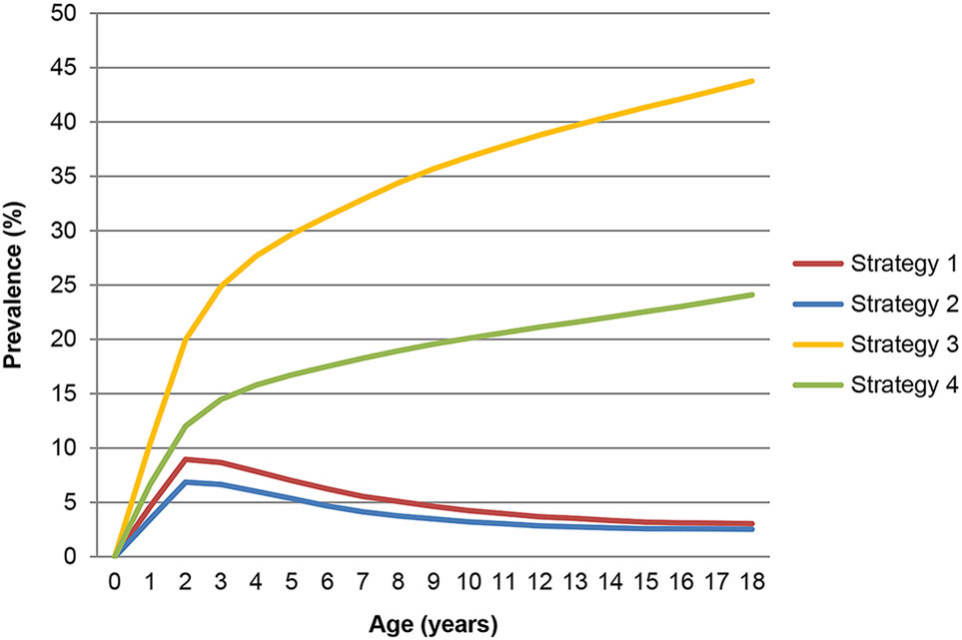
Prevalence by age for atopic eczema

According to strategy 1 and 2, the point prevalence rises to a maximum at age 2 years of 9.0% and 6.9%, respectively. At age 18 years this prevalence drops to 3.0% and 2.5%, respectively. However, if the disorder is considered to be chronic for research purposes, based on strategy 3 and 4 the lifetime cumulative incidences at age 18 years ranges from 24.0–43.8% (Fig. 1).

### Prevalence of asthma

**Fig. 2:**
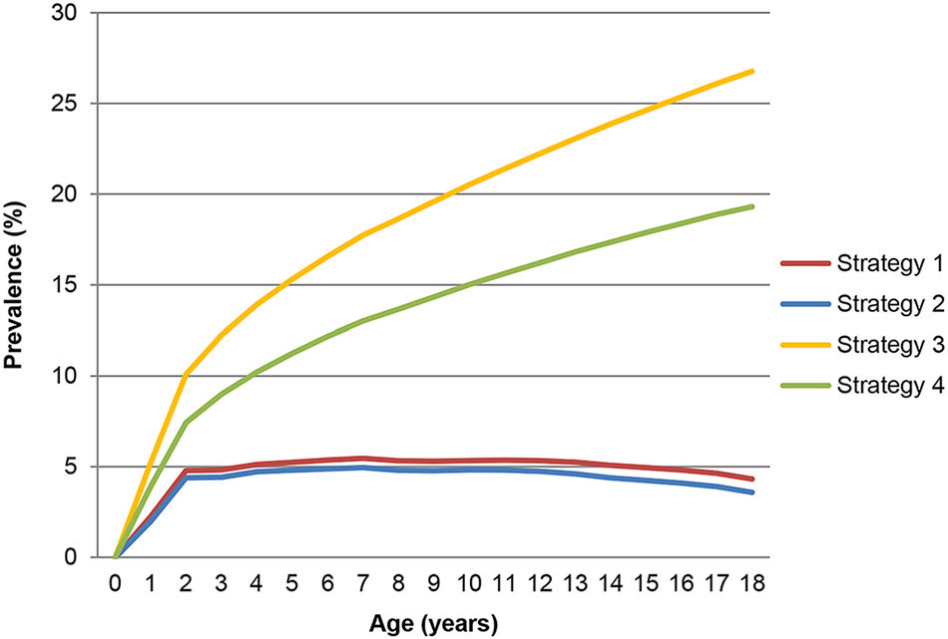
Prevalence by age for atopic asthma

The point prevalence of asthma shows a steep rise in the first two years of life with a maximum prevalence at age 7 years according to strategy 1 (5.5%) and strategy 2 (4.9%), and drops slightly at age 18 years to 4.3% and 3.6%, respectively. The (for research purposes) calculated lifetime cumulative incidences at age 18 year is 19.3–26.8% (Fig. 2).

### Prevalence of allergic rhinitis

**Fig. 3:**
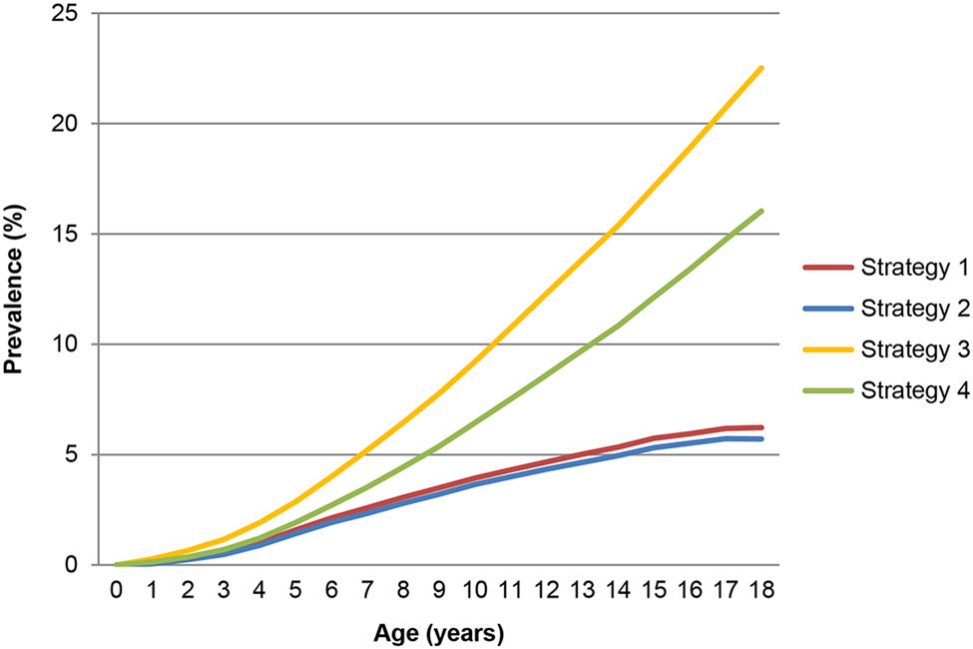
Prevalence by age for allergic rhinitis

In contrast to eczema and asthma, allergic rhinitis shows a relatively consistent rise in prevalence over the years. For strategy1 and 2 the maximum prevalence at age 18 years is 6.2% and 5.7%, respectively. Assuming allergic rhinitis to be a chronic disorder for research purposes, the lifetime cumulative incidence also reaches its maximum at age 18 years, but is substantially higher, i.e., 16.0–22.5% (Fig. 3).

### Prevalence of atopic triad

**Fig. 4:**
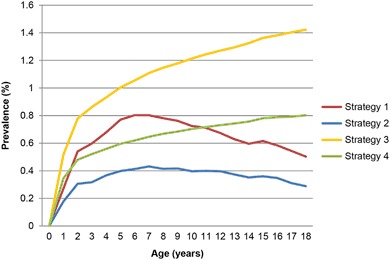
Prevalence by age for atopic triad

The atopic triad is estimated for research purposes. Depending on the strategy used, the maximum prevalence for strategy 1 (0.8%) is reached at age 6 years and that for strategy 2 (0.4%) at 7 years. Both scenarios show a decrease resulting in a point prevalence at age 18 years of 0.5% and 0.3%, respectively. For all four strategies, a maximum prevalence of 1.4% is observed (Fig. 4).

### Interrelationship between the atopic disorders

**Fig. 5:**
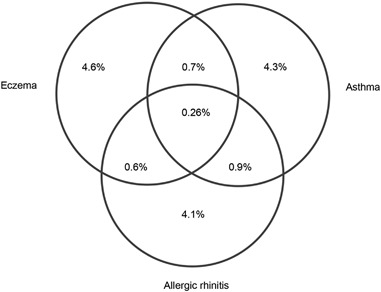
Venn diagram of the overall prevalence (total population: 478,076 children)

Interrelationships between atopic disorders are well known. Of the 478,076 children, based on strategy 228,946 children (6.1%) had eczema, 29,182 (6.1%) had asthma, and 28,064 (5.9%) had allergic rhinitis. Only 1251 (0.26%) children had all three atopic disorders. This is a 12-fold higher prevalence than could be expected by chance (0.022%) based on the three prevalences of the individual atopic disorders. In total 21,862 children had eczema only, 20,382 children had asthma only, and 19,835 children had allergic rhinitis only and no other atopic comorbidity. Of all children with asthma, 19.2% also had allergic rhinitis (Fig. 5).

## Discussion

### Main findings

To retrieve more relevant data from primary care databases, four different strategies were explored. Based on the results of this study, strategy 2, which at least selects cases with potentially more clinically relevant disorders and does not assume that a child will have the disorder for life, seems preferable when interested in the current burden of atopic disorders. Of the 478,076 children finally included, after applying strategy 2, 6.1% had eczema, 6.1% had asthma and 5.9% had allergic rhinitis; these annual point prevalences are in accordance with those found in a recent systematic review.^5^ Only 0.26% children had all three atopic disorders; this is a 12-fold higher prevalence than could be expected by chance based on the three individual prevalences of the atopic disorders (0.022%). This phenomenon was recently described in a meta-analysis^3^ and supports the hypothesis that there could be a fourth distinct group of atopic children that have all three disorders, i.e., they may have their own unique characteristics.

### Interpretation of findings in relation to previously published work

Showing the data simply as recorded in the GP’s database (strategy 1) will result in a risk of overestimation. A possible solution was offered in the literature by applying two selection criteria, i.e., at least two relevant consultations and at least two relevant prescriptions. When applying these criteria, the annual point prevalences only dropped slightly (as expected), but potentially show more clinically relevant cases. The results now more closely approach the annual point prevalences reported in the literature.^5^ However, ideally a gold standard is needed to identify atopic children. Such a gold standard could probably be the evidence of sensitization by specific IgE.^10^ Checking specific IgE is now a requirement of assessment of the patient with asthma. When studying the observed differences between annual point prevalence and cumulative life-time prevalence, a greater understanding of the natural course of these atopic disorders is required. In Germany, Illi et al.^11^ studied the natural course of atopic dermatitis in a cohort of 1314 children from the general population, until age 7 years. The prevalence increased to 21.5% at 2 years of age, but 43.2% were in complete remission by age 3 years. Regarding asthma, Jenkins et al.^12^ screened 7-year-olds for this condition. The study was repeated 25 years later in a random sample (*n* = 750); a quarter of those who had asthma as a child, reported asthma in adulthood. According to Sears, about half to two-thirds of the children with asthma recover.^13^ An explanation for this observed recovery could be that viral infections are the main cause of wheeze before the age of six rather than allergic asthma. This is supported by data from a different Dutch primary care study, which showed that for those children diagnosed with asthma between the age of 0–4 years,  ≥ 60% were no longer known as such by the GP after 2 years, and after 10 years 80% no longer carried this diagnosis.^14^ When the same children were screened for asthma at a later age (10–23 years), 45% still had asthma.^15^ Finally, regarding allergic rhinitis, a prospective study on the course of hay fever in 738 individuals (with an average follow-up of 23 years) showed that in a majority of the adult patients the symptoms of hay fever reduce over the years.^16^ Another prospective study (*n* = 257, mean follow-up to 8 years) on various forms of allergic rhinitis (confirmed by the presence of specific IgE to pollen, pets, or dust mites), looked at the percentage of patients with complete remission of symptoms.^17^ This study found complete remission of symptoms in 12% of patients with pollen allergy, in 19% of patients with an allergy to pets, and in 38% of patients with house dust allergy. The third and fourth strategy assumed that a child would have the atopic disorder for life, resulting in cumulative life-time prevalences that are substantially higher than those reported in the literature.^5^ Based on all the available evidence, it seems incorrect to conclude that atopic disorders are by definition chronic and, therefore, we consider strategies 3 and 4 to be less reliable and are not recommended. Even though the underlying assumptions made for strategies 3 and 4 are not realistic, the differences found between strategy 2 and 4 nevertheless provide an estimation of the number of children that show complete reduction of symptoms. This results in remission rates of 84%, 68%, and 43% at age 10 years and 90%, 81%, and 64% at age 18 years for eczema, asthma, and allergic rhinitis, respectively.

### Strengths and limitations of this study

For the present investigation, we used an extensive and representative primary care database; the number of included cases gives this study substantial power. The potential for using primary care databases of routinely collected clinical data for epidemiology and health policy is, therefore, enormous. However, to use this potential, sound methodologies are needed to turn the huge amount of raw data into meaningful information. An easy to apply strategy is presented in this study to select potentially more clinical relevant cases.

Unfortunately, there is an important limitation. The present study is based on the assumption that the relevant International Classification of Primary Care (ICPC) codes are not missed. For example, a child that has ICPC code R03 (wheezing) and regularly uses inhalation corticosteroids probably has asthma. However, when the child is not coded correctly as having R96 (asthma), or is not coded at all, it will not be possible to identify this child as having asthma. To include this child as an asthmatic patient, a new or adjusted episode R96 needs to be created by the researcher. Although this is a complex problem, there are different ways to deal with it. The most sensitive methodis to study the complete EMR of the individual patient; unfortunately, this is very time consuming and raises privacy issues. Another option is to use computer software that analyses free text; however, the accuracy of this method is determined by the quality of the script used. A faster and probably more consistent way of identifying a child, is to use ‘‘templates’’ that are based on a combination of routinely and standardized coded data from EHRs such as standardized measurements, ICPC-coded comorbidity, and Anatomical Therapeutic Chemical (ATC)-coded prescriptions. According to a recent study,^18^ based on general practice data, children diagnosed with asthma can be reliably identified with a range of medication proxies (sensitivity 54% and positive predictive value 84%). However, the use of prescription data for the identification of children diagnosed with eczema and allergic rhinitis is more problematic; one reason for this is that (some) reliever medication is freely available over the counter. Comorbidity data could also be used as a source to identify misclassified children. However, although many studies have shown a relationship between different comorbidities and atopic disorders, to our knowledge no study has used comorbidity to identify atopic disorders.

Food allergies are also closely associated with atopic disorders. Unfortunately, in this study it was not possible to reliably analyze food allergies, since the ICPC-1 coding system does not have specific codes for food allergies.

### Implications for future research, policy, and practice

The results of this study emphasize the importance of better coding. Further research is needed to create proxies based on standardized coded variables to identify atopic disorders in order to address the risk of underestimation. Some attempts have been made, such as Asthma Critic (a decision-support system for asthma and chronic obstructive pulmonary disease),^19^ which aims to generate patient-specific feedback based on routinely recorded data in EMRs. In order to address the risk of overestimation, future clinical guidelines should also include criteria that help physicians to identify atopic diagnoses, which are no longer clinically relevant.

In the future, research using extensive databases will become more popular due to their increased availability. Epidemiological studies on atopic disorders are reaching the limit of what can be achieved through conventional hypothesis-driven research.^20^ This new era of ‘‘big data’’ allows smarter and more powerful statistical analysis, especially when analyzing metadata. Future collaborative analysis could also facilitate interdisciplinary dialogue between clinicians and scientists.

## Conclusions

In conclusion, research using extensive databases will become more popular due to their increased availability; we are now in the era of ‘‘big data’’. Future collaborative (meta) analysis on the valid use of routinely recorded clinical data from big databases is needed in order to be able to develop valid search strategies to identify atopic children. This study contributes to a better understanding of the use of primary care data. Based on the results of this study, strategy 2, which at least corrects for the risk of overestimation due to misclassification and does not assume that a child will have the disorder for life, seems preferable and can easily be applied. The limitations of primary care data that result in underestimation are more challenging, since some patients are also able to self-manage their disorder. Studies are required to create proxies based on routinely recorded and standardized clinical coded data that can help identify atopic disorders that are missed or misclassified.

## Methods

### Study population

NIVEL-PCD is based on routinely recorded data in EHRs of all listed patients in the participating practices. In 2014, about 500 general practices participated, including data of about 1,700,000 patients (www.nivel.nl/en/dossier/nivel-primary-care-database). EHR data include a variety of information regarding type of consultation, morbidity, and prescriptions. Data were available from 2002–2014 and are representative for the Dutch population.^21^ Primary care physicians recorded morbidity using the ICPC-1. The ICPC is a classification method for primary care encounters and is accepted by the WHO.^22^ Dutch GPs cluster relevant consultations, prescriptions, and referrals in ICPC classified episodes of care.

For the present study, we only used morbidity data from the EHRs of general practices with sufficient data quality, fulfilling the following criteria: at least 500 listed patients (standard practice: 2350 patients), complete morbidity registration (defined as  ≥ 46 weeks/year), and sufficient ICPC coding of diagnostic information (defined as  ≥ 70% of the recorded disease episodes labeled with an ICPC code).

### Selection of atopic children

From the general practices in NIVEL-PCD, all listed children (aged 0–18 years) with sufficient data (in the period 2002–2014) were selected. For each child, a minimum follow-up of 3 years was required to reduce the risk of registration bias. According to NIVEL, Dutch GPs see about 77% of their patients at least once a year;^23^ therefore, a 3-year follow-up allows the GP sufficient time to diagnose a child with atopic disorders. Follow-up ends when a child would change to a GP that is not working in a NIVEL-PCD clinic, or when the child would have died. For these children, the following descriptive data were routinely collected: period in which the individual child was registered in the clinic, unique code of the GP practice, sex, and year and quarter of birth. For all these children, ICPC-coded episodes regarding atopic eczema (S87), asthma (R96), and allergic rhinitis (R97) were extracted when applicable with their starting and closing dates.

### Episode (re)construction

At each new encounter in general practice, a Dutch GP starts a new episode of care. If the patient returns to the GP for the same disorder, or when the patient orders (repeat) medication relevant to that disorder, it should be recorded as a follow-up contact within that specific episode of care.

In the present study, four different strategies were examined with the aim to obtain a better understanding of prevalence estimates based on primary care data: two strategies are related to the beginning of an episode of care and the other two are related to the ending of an episode of care. Table 1 presents a summary of the strategies.

**Table 1:** Summary of the four strategies examined

Strategy 1	Presents the prevalence based on the recorded episodes of care
Strategy 2	Presents the prevalence based on corrected episodes of care (by applying selection criteria: at least two relevant consultations and at least two relevant prescriptions)
Strategy 3	Presents the prevalence based on the recorded episodes of care, but the disorders are considered to be chronic
Strategy 4	Presents the prevalence based on corrected episodes of care (by applying selection criteria from strategy 2), but the disorders are considered to be chronic

Concerning the start of an episode, either the episodes of care were used as recorded in the database and one accepts the risk of *overestimation* due to working with ‘‘probability diagnoses’’, or these episodes of care were corrected by applying selection criteria, focusing on cases with higher probability of a clinical relevant disorder (see below). With respect to the ending of an episode of care, two identical strategies were applied. Either the episodes of care were used as recorded or these episodes of care were corrected by extending the closing date, assuming that atopic disorders were chronic.

#### Start of an episode of care

Strategy 1 uses the episode of care as recorded in the EHRs of the GP and accepts a risk of overestimation. In the second strategy (strategy 2), correcting for a possible overestimation, different selection criteria were taken into consideration based on our previous review.^5^ Using these criteria, ICPC codes and their related episodes of care can be corrected, reducing the risk of misclassification and selecting cases with a higher probability of a clinical relevant disorder. For example, if a GP suspects that a child has asthma and labels the encounter accordingly with R96, this can later be corrected as not having asthma if this child never visits the GP again for this problem or never receives the appropriate medication. In practice, this implies the following requirements: at least two episode-related contacts (either consultations, home visits, telephone calls, or prescriptions) and a minimum of two relevant prescriptions had to be prescribed. The ATC Classification System was used to identify relevant prescriptions. For eczema the ATC code D07 (dermatological corticosteroids) was used, for asthma the ATC code R03 (drugs for obstructive airway diseases) was used, and for allergic rhinitis the ATC codes R01AC (nasal preparation of antiallergic agents, excl. corticosteroids), R01AD (nasal preparation of corticosteroids) and R06 (antihistamines for systemic use) were used. These medication proxies have been tested by Mulder et al.^18^ Since some EHRs do not routinely link relevant prescriptions in the correct episodes, all recorded prescriptions in the EHRs were studied. When a patient could not meet the criteria of having at least two contacts and two relevant prescriptions, the patient is considered to be a child in the ‘‘population at risk’’.

#### Closure of an episode of care

In the present study, two strategies (3 and 4) considered the atopic disorders to be chronic for research purposes. Since data is available for all patients in our database regarding the first date on which a diagnosis was made (each child could be incident only once in its life), it is possible to determine the number of children diagnosed at each year and for each age. When adding these annual numbers for the consecutive years of interest, one in fact calculates a cumulative incidence. Since no data is missing regarding the first date of the disorder, this cumulative incidence will approximate a cumulative life-time prevalence. Strategy 3 shows the cumulative incidences based on strategy 1, and in strategy 4 it is based on strategy 2.

#### Atopic triad

Finally, ‘‘atopic triad’’ episodes were created for research purposes, based on a suggestion reported in a meta-analysis.^3^ Such an episode was only created when a child was diagnosed with all three atopic disorders. The first date when a child was diagnosed with at least one of the disorder, is considered the starting date of the ‘‘atopic triad’’ episode. The closing date of the episode is equal to the last contact date recorded for one of the atopic disorders.

### Statistical analyses

Annual point prevalence rates were calculated as percentages on the first of January for each age (0–18 years). The denominators for the calculations were also determined on this date. Cumulative life-time prevalences, based on the assumption that the disorder is chronic, are based on the cumulative incidences (strategy 3 and 4). This cumulative incidence equals a life time prevalence, since the complete medical history of a patient is available in the EHRs. To calculate the interrelationships between the atopic disorders, for every child’s EHR with sufficient data quality and at least 3 years of follow-up, it was determined whether he/she had one or more atopic disorders or not, in the period from 2002–2014. All calculations were conducted in Stata 13 and Excel 2010.

### Ethical approval

Dutch law allows the use of anonymous EHR data for research purposes under certain conditions. According to this legislation, it is not necessary to obtain informed consent from patients or approval from a medical ethics committee for this type of observational study that contains no directly identifiable data (Dutch Civil Law, Article 7: 458). Therefore, no waiver of ethical approval was obtained from an Institutional Review Board or ethics committee. The authors did not have access to identifying information at any moment during the analysis of the data.

## Electronic supplementary material

Appendix 1
