# Supplementary figures and images for: Reliably estimating prevalences of atopic children: an epidemiological study in an extensive and representative primary care database
Source: NPJ Prim Care Respir Med. 2017 Apr 13;27:23. doi: 10.1038/s41533-017-0025-y (PMC5435092; doi:10.1038/s41533-017-0025-y)

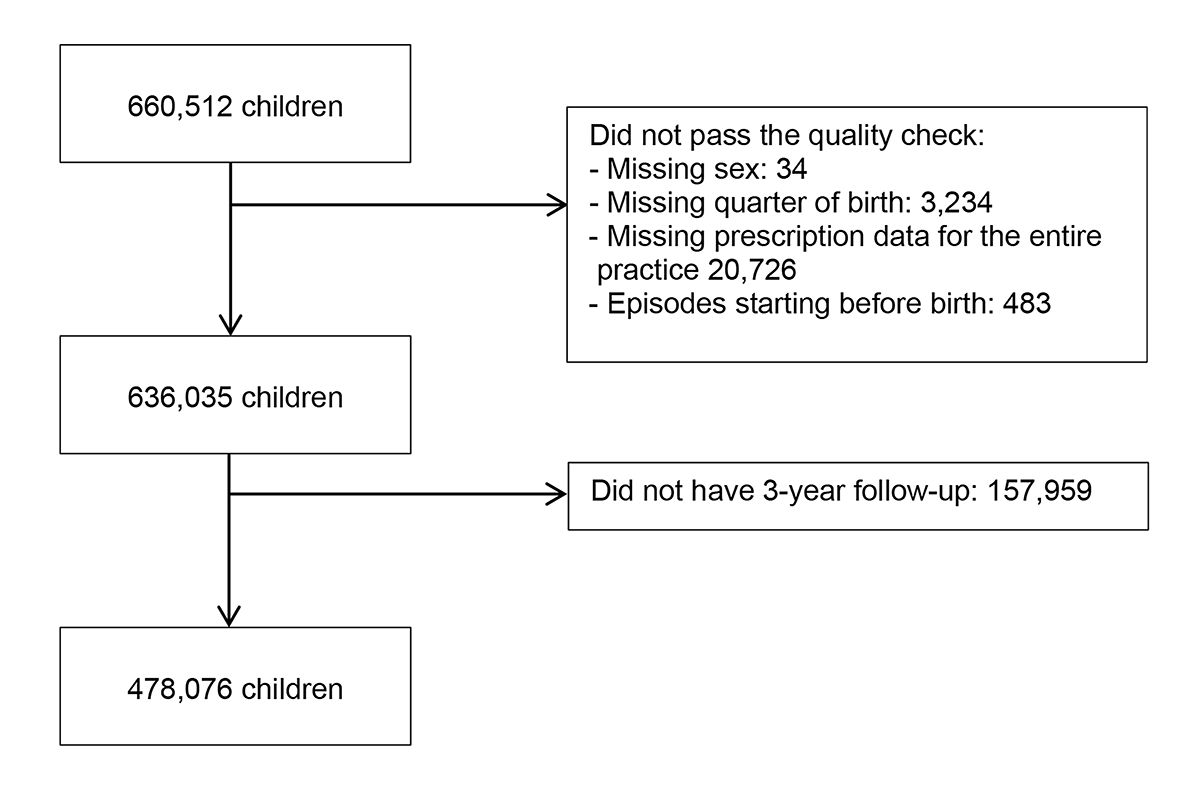

Supplement: Supplementary file 1 — Appendix 1 [file 41533_2017_25_MOESM1_ESM.tif]
